# Lignans from *Bursera fagaroides* Affect In Vivo Cell Behavior by Disturbing the Tubulin Cytoskeleton in Zebrafish Embryos

**DOI:** 10.3390/molecules24010008

**Published:** 2018-12-20

**Authors:** Mayra Antúnez-Mojica, Andrés M. Rojas-Sepúlveda, Mario A. Mendieta-Serrano, Leticia Gonzalez-Maya, Silvia Marquina, Enrique Salas-Vidal, Laura Alvarez

**Affiliations:** 1 CONACYT-Centro de Investigaciones Químicas-IICBA, Universidad Autónoma del Estado de Morelos, Cuernavaca 62209, Morelos, Mexico; myam@uaem.mx; 2 Facultad de Ciencias, Universidad Antonio Nariño, Armenia Quindío 63003, Colombia; andres.rojas@uan.edu.co; 3 Departamento de Genética del Desarrollo y Fisiología Molecular, Instituto de Biotecnología, Universidad Nacional Autónoma de México, Cuernavaca 62210, Morelos, Mexico; mbimams@nus.edu.sg; 4 Facultad de Farmacia, Universidad Autónoma del Estado de Morelos, Cuernavaca 62209, Morelos, Mexico; letymaya@uaem.mx; 5 Centro de Investigaciones Químicas-IICBA, Universidad Autónoma del Estado de Morelos, Cuernavaca 62209, Morelos, Mexico; smarquina@uaem.mx

**Keywords:** *Bursera fagaroides*, podophyllotoxin-type lignans, cell cycle, cell migration, epiboly, microtubules, F-actin, cancer, lignans

## Abstract

By using a zebrafish embryo model to guide the chromatographic fractionation of antimitotic secondary metabolites, seven podophyllotoxin-type lignans were isolated from a hydroalcoholic extract obtained from the steam bark of *Bursera fagaroides*. The compounds were identified as podophyllotoxin (**1**), β-peltatin-A-methylether (**2**), 5′-desmethoxy-β-peltatin-A-methylether (**3**), desmethoxy-yatein (**4**), desoxypodophyllotoxin (**5**), burseranin (**6**), and acetyl podophyllotoxin (**7**). The biological effects on mitosis, cell migration, and microtubule cytoskeleton remodeling of lignans **1**–**7** were further evaluated in zebrafish embryos by whole-mount immunolocalization of the mitotic marker phospho-histone H3 and by a tubulin antibody. We found that lignans **1**, **2**, **4,** and **7** induced mitotic arrest, delayed cell migration, and disrupted the microtubule cytoskeleton in zebrafish embryos. Furthermore, microtubule cytoskeleton destabilization was observed also in PC3 cells, except for **7**. Therefore, these results demonstrate that the cytotoxic activity of **1**, **2,** and **4** is mediated by their microtubule-destabilizing activity. In general, the in vivo and in vitro models here used displayed equivalent mitotic effects, which allows us to conclude that the zebrafish model can be a fast and cheap in vivo model that can be used to identify antimitotic natural products through bioassay-guided fractionation.

## 1. Introduction

Lignans are a big family of secondary metabolites biosynthesized in plants through the shikimic acid pathway that represent an important class of compounds in cancer research studies [1,2]. One of the most representative groups of lignans are the aryltetralin lignans [3]. Into this group of compounds, podophyllotoxin (**1**) is the most known because of its use as an effective antiviral for genital warts treatment. Podophyllotoxin inhibits tubulin polymerization inducing the mitotic arrest of cancer cells [4]. In vitro, it binds to the colchicine binding site [5,6]. Because of the unacceptable gastrointestinal toxicity, many semisynthetic podophyllotoxin derivatives were developed and tested for anticancer activity [7]. Etoposide and teniposide are glycosylated derivatives of podophyllotoxin developed in the 1970s. These derivatives are used in many clinical chemotherapeutic regimens, because of their easy administration and good toleration. However, the action mechanism through which they exert their effect is different from that of podophyllotoxin (**1**), since these semisynthetic drugs break DNA by the interaction with the topoisomerase II enzyme [1].

**Figure 1:**
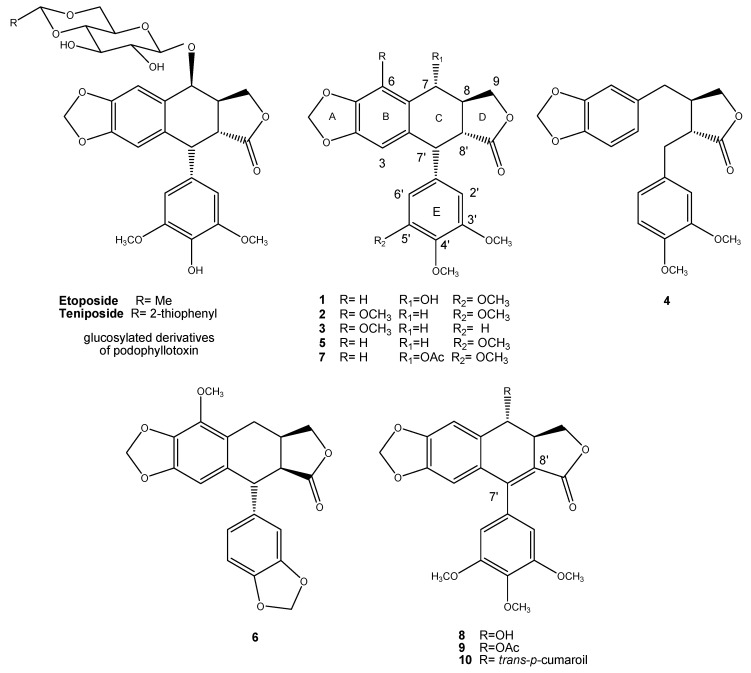
Glycosylated derivatives of podophyllotoxin (etoposide and teniposide) and podophyllotoxin-type lignans **1**–**10:** podophyllotoxin (**1**), β-peltatin-A-methylether (**2**), 5′-desmethoxy-β-peltatin-A-methylether (**3**), desmethoxy-yatein (**4**), desoxypodophyllotoxin (**5**), burseranin (**6**), acetyl podophyllotoxin (**7**), 7′,8′-dehydropodophyllotoxin (**8**), 7′,8′-dehydroacethyl podophyllotoxin (**9**), and 7′,8′-dehydro *trans-p*-cumaroylpodophyllotoxin (**10**), isolated from the steam bark of *Bursera fagaroides*.

Our research team has characterized an interesting traditional Mexican medicinal plant named *Bursera fagaroides*, which is popularly used as an antitumoral. Our previous studies described the isolation of seven aryltetralin-type lignans including podophyllotoxin (**1**), β-peltatin-A-methylether (**2**), 5′-desmethoxy-β-peltatin-A-methylether (**3**), desmethoxy-yatein (**4**), desoxypodophyllotoxin (**5**), burseranin (**6**), and acetyl podophyllotoxin (**7**) [8], as well as three aryldihydronaphtalene-type lignans, i.e., 7′,8′-dehydropodophyllotoxin (**8**), 7′,8′-dehydroacethyl podophyllotoxin (**9**), and 7′,8′-dehydro *trans-p*-cumaroylpodophyllotoxin (**10**) (Figure 1) [9]. The cytotoxic evaluation of these compounds indicated that all lignans showed potent cytotoxic activity against a panel of four human cancer cell lines (KB, HF-6, MCF-7, and PC-3), being selective against prostate cancer cells (PC-3). 

Other studies proved that lignans **3** and **7** are more potent than the semisynthetic podophyllotoxin drug etoposide (14.1 and 7.6 µg/mL, respectively) in the human breast cancer cell line BT-549 [10]. Additionally, compounds **3**, **6**, and **7** showed anti-giardial activity in vitro, provoking morphological alterations in *Giardia* parasite [11]. In a deeper molecular recognition study of lignans **3**, **7**, and **9**, the action mechanism consisting in disrupting microtubule networks and cell cycle arrest in G2/M phase in the human carcinoma lung cell line A549 was demonstrated. Also, we established that these compounds interact with the tubulin colchicine binding site with a high binding constant (K_b_) [6].

Recently, in vivo studies performed with the aryldihydronaphtalene-type lignans (**8**–**10**) in the developing zebrafish embryo model, demonstrated that these compounds promote mitotic arrest, delay cell migration and disrupt microtubules [9]. 

Continuing the study of this important medicinal plant, in this work, we analyzed the hydroalcoholic extract of *B. fagaroides* steam bark, employing developing zebrafish embryos as a model with the aim to identify compounds able to affect the tubulin cytoskeleton and to find a faster and easier biological model to discover new destabilizing anticancer drugs. 

## 2. Results and Discussion 

### 2.1. In Vivo Analysis of Mitotic Arrest in Zebrafish Embryos 

Histone H3 phosphorylated at serine 10 (H3S10ph) has long been used to identify mitotic cell nuclei in cultured cell lines [12] as well as in whole embryos such as those of *Xenopus* and zebrafish [13,14,15]. In particular, 24 h-old zebrafish embryos have been used to screen the effects of small chemical molecules on the cell cycle and the induction of mitotic arrest using whole-mount immunohistochemistry of H3S10ph [16]. 

First, we evaluated the effect of a *B. fagaroides* hydroalcoholic extract (HA) on mitotic arrest in whole 24 h post fertilization (hpf) zebrafish embryos. Nocodazole and aphidicolin were used as controls of inhibition of DNA polymerase and tubulin depolymerization, respectively [17,18]. The aphidicolin-treated embryos exhibited considerably fewer H3S10ph-positive nuclei compared with the control embryos incubated with the vehicle (DMSO). In contrast, nocodazole-treated embryos appeared to have substantially more H3S10ph-positive nuclei compared with the control embryos (Figure S1).

**Figure 2:**
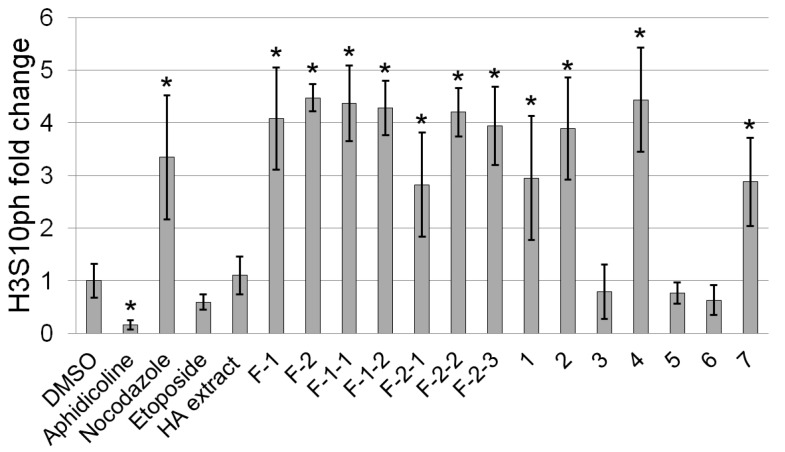
Staining of mitotic nuclei by phospho-histone H3 (H3S10ph) in zebrafish embryos treated with the hydrocoholic extract fractions and the pure compounds: Podophyllotoxin (**1**), β-peltatin-A-methylether (**2**), 5′-desmethoxy-β-peltatin-A-methylether (**3**), desmethoxy-yatein (**4**), desoxypodophyllotoxin (**5**), burseranin (**6**), and acetyl podophyllotoxin (**7**). The fold changes were determined in comparison with the control (DMSO-treated). Values represent means ± s.d. * Significant differences (*p* < 0.001). These results are also shown in Table S1.

The HA extract of *B. fagaroides* did not affect the number of mitotic cells, as measured by the number of H3S10ph-positive nuclei with respect to that of the control (Figure 2, Figure S2, and Table S1). Nevertheless, in our previous study, this extract showed potent in vitro cytotoxic activity and strong in vivo antitumor activity against L5178Y lymphoma in mice [8]. Therefore, we considered that the lack of effect of the HA extract in zebrafish embryos could be due to poor bioavailability because of the low concentration of the active compounds present in the extract. For this reason, we decided to test the effect of its chromatographic fractions. Therefore, successive chromatographic fractionations of the HA extract were performed, which afforded four fractions, two of which (F-1 and F-2) were analyzed (Figure S2). These two fractions were the most active when the fractionation was guided by the cytotoxic activity. In contrast, F-3 and F-4 were not evaluated in this work because of their lack of cytotoxic activity reported by Rojas et al. [8]. Fractions F-1 and F-2 increased the number of H3S10ph-positive nuclei by 4.08- and 4.48-fold, respectively (Figure 2 and Table S1). The treated embryos that exhibited increased H3S10ph levels also displayed abnormal morphology; these embryos were curved ventrally (Figure S2), like the embryos treated with nocodazole. 

Chromatographic purification of fractions F-1 and F-2 afforded seven lignans identified as podophyllotoxin (**1**), β-peltatin-A-methylether (**2**), 5′-desmethoxy-β-peltatin-A-methylether (**3**), desmethoxy-yatein (**4**), desoxypodophyllotoxin (**5**), burseranin (**6**), and acetyl podophyllotoxin (**7**) by direct comparison with authenticated samples obtained in previous work (Figure 1) [8]. 

**Figure 3:**
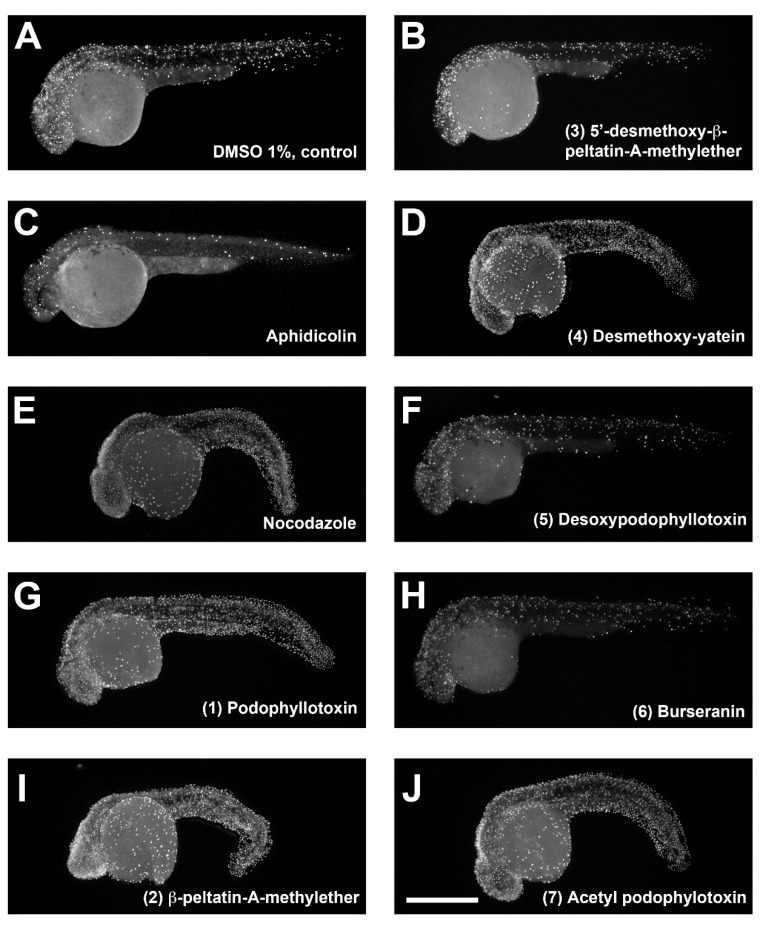
Whole-mount immunolocalization of H3S10ph in zebrafish embryos treated with *B. fagaroides* lignans. Wild-type 24 h post-fertilization zebrafish embryos were immunostained for H3S10ph after a 6 h treatment with different compounds. (**A**) Dimethyl sulfoxide (control), (**B**) Aphidicolin, (**C**) Nocodazole, (**D**) Podophyllotoxin (**1**), (**E**) β-peltatin-A-methylether (**2**), (**F**) 5′-desmethoxy-β-peltatin-A-methylether (**3**), (**G**) Desmethoxy-yatein (**4**), (**H**) Desoxypodophyllotoxin (**5**), (**I**) Burseranin (**6**), (**J**) Acetyl podophyllotoxin (**7**). The images were visualized by confocal microscopy. Scale bar, 500 μm.

Next, we analyzed the effect of all the isolated podophyllotoxin-type lignans (**1**–**7**) on the H3S10ph marker and morphology in zebrafish embryos (Figure 3). The results showed that compounds **1**, **2**, **4,** and **7** significantly increased the H3S10ph levels, by 2.95-, 3.89-, 4.44- and 2.88-fold, respectively (Figure 2 and Table S1).

In contrast, compounds **3**, **5,** and **6** did not affect the number of mitotic cells or the circularity of the embryos. Zebrafish embryos treated with podophyllotoxin (**1**) exhibited H3S10ph levels 2.95-fold higher than those of the control embryos (Figure 2). Many semisynthetic derivatives of podophyllotoxin with anticancer activity have been described, such as etoposide. We asked whether etoposide had any effect in this model. The results revealed that etoposide appeared to slightly decrease the number of H3S10ph-positive nuclei compared with control embryos, but this difference was not statistically significant (Figure 2 and Table S1). 

This difference could be attributed to the mechanism of action by which these compounds act. It is well known that etoposide interacts with DNA and inhibits DNA-topoisomerase II, while podophyllotoxin (**1**) and its congeners (**3**, **6**, and **7**) inhibit tubulin assembly [6].

### 2.2. Characterization of Compounds that Affect Cell Migration in the Zebrafish Model

**Figure 4:**
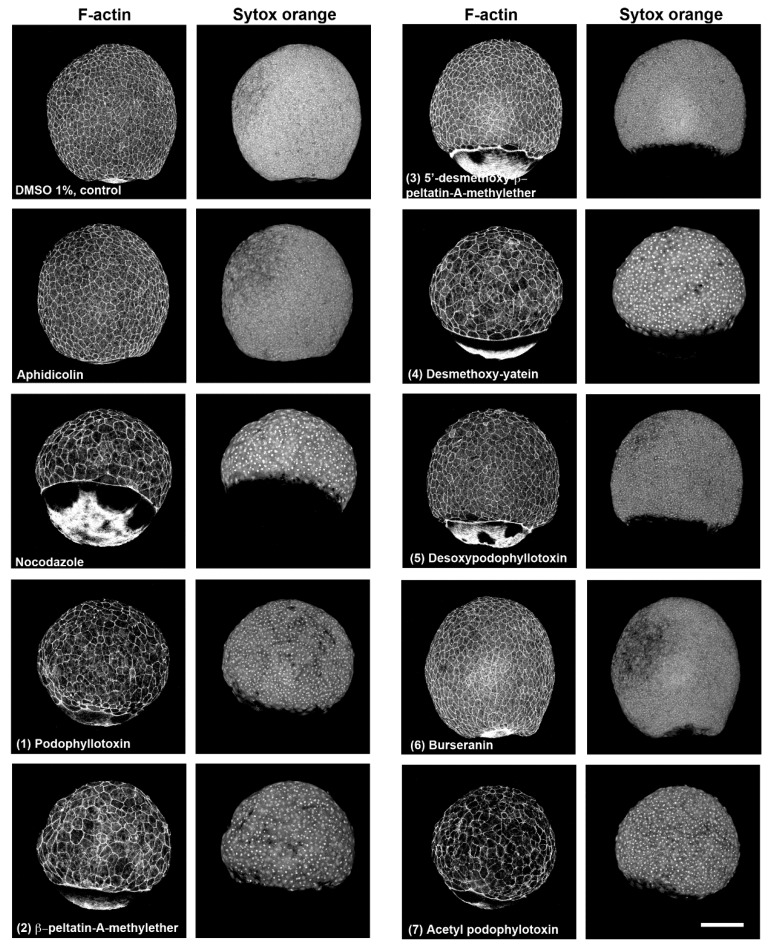
Immunolocalization of actin filaments and nuclei in zebrafish embryos after treatment with lignans (**1**)–(**7**), dimethyl sulfoxide (negative control), Aphidicolin, and Nocodazole (positive controls). Sphere-stage zebrafish embryos were treated with different compounds until control embryos reached 90% epiboly, fixed, processed for actin and nuclei staining, and visualized by confocal microscopy. Scale bar, 250 μm.

Previous studies have shown that when the microtubule cytoskeleton is compromised in early zebrafish embryos by ultraviolet light radiation or by nocodazole treatment, epiboly migration is severely affected [19,20]. The effect on epiboly migration has been used to screen libraries of compounds that destabilize microtubules [21]. By using this approach, we analyzed the effect of pure lignans (**1**–**7**) on epiboly migration in zebrafish embryos. Embryo treatment started at the sphere stage, and all treatment groups were fixed at the same time point when the control DMSO-treated embryos reached 90% of epiboly. Compounds **1**, **2**, **4,** and **7** induced epiboly delay and larger blastoderm cells and nuclei, as evidenced by phalloidin alexa and SYTOX green staining of the embryos (Figure 4). These effects show similarities to those of nocodazol treatment, although this destabilizing microtubule compound showed a more penetrant phenotype. Compounds **3** and **5** delayed epiboly too but did not show effects on the size of blastoderm cells or in their nuclei (Figure 4). 

### 2.3. In Vivo and In Vitro Analysis of Compounds that Affect Microtubules 

To corroborate that these lignans possess microtubule destabilizing activity, we decided to perform a tubulin immunolocalization assay in embryos treated with lignans **1**–**7**. Starting from the sphere stage, embryos in all treatment groups were fixed when they reached 50% of epiboly. This stage was chosen to perform tubulin immunolocalization since the yolk cells present large and conspicuous microtubules that are readily visualized by confocal microscopy [22].

**Figure 5:**
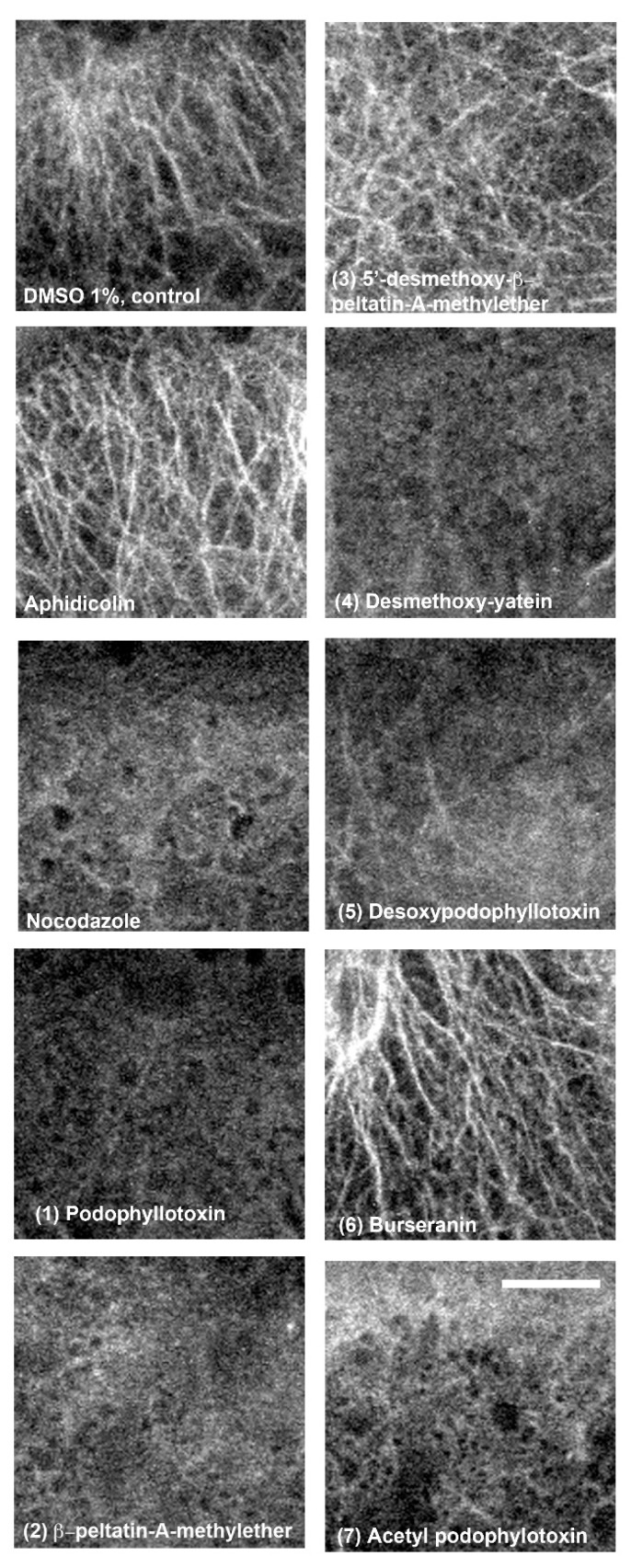
Whole-mount immunolocalization of yolk cell microtubules in zebrafish embryos after treatment with lignans (**1**)–(**7**). Sphere-stage zebrafish embryos were treated with different compounds until all embryos reached 50% epiboly; they were then fixed, processed for microtubule fluorescent immunolocalization, and visualized by confocal microscopy. Scale bar, 25 μm.

The results showed that, again, compounds **1**, **2**, **4,** and **7** induced microtubules destabilization similar to nocodazol (Figure 5). On the other hand, zebrafish embryos treated with compound **3** showed some degree of disorganized microtubules, and compound **5**-treated embryos presented less abundant arrays of microtubules, although neither treatment showed the degree of microtubule destabilization found in embryos treated with compounds **1**, **2**, **4,** and **7** (Figure 5). 

**Figure 6:**
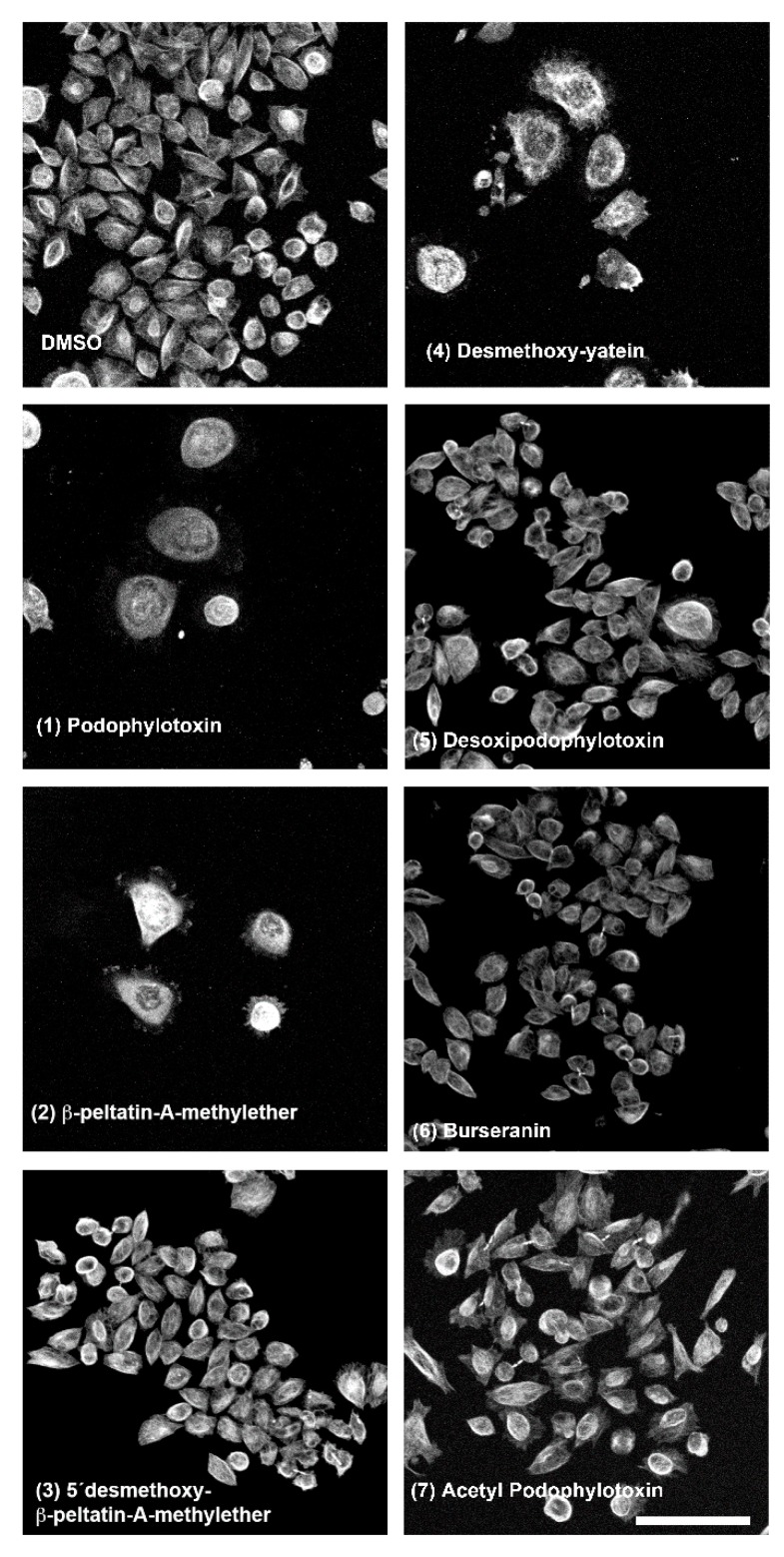
Whole-mount immunolocalization of cancer PC3 cells microtubules as viewed by confocal microscopy after treatment with DMSO (control) and lignans (**1**)–(**7**). Scale bar, 100 µm.

Finally, we asked if the identified lignans also destabilize the microtubule cytoskeleton in cancer cells when exposed in vitro to these compounds. Specifically, we tested the effect of the pure lignans **1**–**7** in the PC3 human prostate cancer cell line, as this was the most susceptible cancer cell line when evaluated by Rojas et, al. [8]. We found that **1**, **2,** and **4** destabilized microtubules; in contrast **3**, **5**, **6**, and **7** did not affect the microtubule cytoskeleton (Figure 6). 

These results indicated that six of the seven *B. fagaroides* lignans showed equivalent effects to those observed in whole zebrafish embryos. Only acetyl podophyllotoxin (**7**) presented a dissimilar effect in both systems, severely affecting microtubules in zebrafish embryos (Figure 4) without showing any effect on the microtubule cytoskeleton of PC3 cells in the assay (Figure 6). 

In general, lignans with major conformational mobility, such as **1**, **2**, **4**, and **7**, isolated in this work, showed a better in vivo effect than the planar lignans **8**–**10** isolated previously [9].

## 3. Materials and Methods

### 3.1. Plant Material, Extraction, and Isolation

The bark of *B. fagaroides* (Kunth) Engl., was collected in the village of Capula between Zacapu and Quiroga, Michoacán, Mexico. Its identification was made in the herbarium of the Instituto Mexicano del Seguro Social (registration number-12 051 IMSSM) and the Institute of Botany, University of Guadalajara (IBUG-140 748). The voucher specimens were deposited in both herbariums. 

The bark of *B. fagaroides* (1700 g) was dried at room temperature, and the dry material was extracted by triplicate with hexane, followed by MeOH/H_2_O 70:30 to yield 40.5 g hydroalcoholic extract (HA). Then, the extract was fractionated by percolation, eluting with mixtures of n-hexane/acetone/MeOH with increasing polarity, to yield: F-1, 1.09 g (8:2:0), F-2, 1.45 g (6:4:0), F-3, 16 g (0:100:0), and F-4, 18.5 g (0:1:1). 

The fractionation of F-1 led to the purification of podophyllotoxin (**1**) (4.92 mg), β-peltatin-A-methylether (**2**) (3.5 mg), and 5′-desmethoxy-β-peltatin-A-methylether (**3**) (7.9 mg), while desmethoxy-yatein (**4**) (8.7 mg), desoxypodophyllotoxin (**5**) (4.3 mg), burseranin (**6**) (15 mg), and acetyl podophyllotoxin (**7**) (6.2 mg) were obtained from F-2. This procedure was performed by RP–HPLC as described by Rojas-Sepulveda et al. [8]. The pure lignans were characterized by direct comparison (^1^H NMR, co-TLC, Table S2) with authenticated samples available in our laboratory [8].

### 3.2. Fish Maintenance and Strains

Zebrafish (*Danio rerio*) embryos were obtained from natural crosses from wild-type and AB–TU–WIK hybrid lines. Embryo stages were determined by morphological criteria [23]. Zebrafish were handled in compliance with the local animal welfare regulations, EU Directive 2010/63/EU indications [24], and zebrafish use was approved by the Ethics Committee of Instituto de Biotecnología, UNAM. 

### 3.3. Chemical Treatment of Zebrafish Embryos

Treatments were performed as previously described [9,16]. In brief, the compounds for the chemical treatments were diluted in anhydrous DMSO (276855, Sigma-Aldrich, Saint Louis, MO, USA). Plant extracts and pure compounds for screening were tested at a standard final concentration of 200 µg/mL in water. The control compounds were aphidicolin (10 µg/mL, A0781, Sigma-Aldrich, Saint Louis, MO, USA) and nocodazole (10 µg/mL, M1404, Sigma-Aldrich, Saint Louis, MO, USA). Etoposide was obtained from Sigma-Aldrich (E1383). To test the effect on mitotic arrest, 10 zebrafish embryos of 24 h of postfertilization age, for each treatment, were incubated at 28 °C in egg water (60 µg/mL of “Instant Ocean” sea salts in distilled water) for 6 h in 48-well plates [25]. Three microliters of each compound stock solution were added to a total volume of 300 µL at the beginning of the incubation. Control embryos were treated with DMSO alone at 1% final concentration (v/v) and processed for immunohistochemistry as described below. To test the effect on cell migration and on F-actin, zebrafish embryos at sphere stage were incubated until the control embryos reached 90% epiboly to bud stage. Finally, to test the effect on the tubulin cytoskeleton, zebrafish embryos at sphere stage were incubated until the control embryos reached 60% epiboly. Embryos in all treatments were stage-matched, fixed, and processed as described below. 

### 3.4. Immunofluorescense and Immunohistochemistry

Whole-mount fluorescent immunostaining against the mitotic marker serine 10 phospho-histone H3 (H3S10ph) in zebrafish embryos was used as previously described [13]. For immunohistochemistry, embryos were processed as for fluorescent immunostaining except that after overnight incubation with the primary anti-phospho-histone H3 antibody, the embryos were processed with R.T.U. Vectastain Universal Quick Kit (PK-7800, Vector, Burlingame, CA, USA) and developed in diaminobenzidine with nickel contrast substrate (SK-4100, Vector, Burlingame, CA, USA). For the analysis of the effects on microtubules, the embryos were incubated with the different compounds, fixed at 60% epiboly, and processed as described. 

The primary antibody used was a mouse anti-α-tubulin monoclonal antibody (T9026, Sigma-Aldrich, Saint Louis, MO, USA), and a goat anti-mouse coupled to Alexa Fluor 647 (A21235, Molecular Probes, Eugene, OR, USA) was used as a secondary antibody. For the analysis of cell migration, all embryos were fixed at the same time when the controls reached the tail bud stage. Afterwards, they were washed three times, stained for 1 h at room temperature with SYTOX orange (S11368, Molecular Probes, Eugene, OR, USA) diluted 1:2000 in blocking buffer to visualize DNA and nuclei, and counter-stained with phalloidin Alexa Fluor 488 (A12379, Molecular Probes, Eugene, OR, USA). The embryos were then washed three times, mounted, and imaged by confocal microscopy. 

### 3.5. Fluorescence Microscopy

Th fluorescent signals corresponding to H3S10ph-positive cells in whole zebrafish embryos where imaged by fluorescence microscopy with a 5× objective, 0.15 N.A. Plan-Neofluar under a Zeiss Axioscop Microscope. Image stack (10 to 14 images per embryo) corresponding to different focal planes were acquired with a CoolSNAP cfd CCD camera (Roper Scientific, Tucson Arizona, AZ, USA), controlled by MicroManager 1.5 software (NIH, Bethesda, MD USA). Image resolution was 1392 × 1040, and stack images were saved in 8-bit multi-image TIFF file.

### 3.6. Confocal Laser Scanning Microscopy

Zebrafish embryos stained with the specified fluorescent dyes were visualized on a Zeiss LSM 510 META confocal inverted microscope (Carl Zeiss, Jena, Germany) with a Plan-Neofluar 10× (0.3 N.A.) objective, a Plan-Neofluar 20× (0.5 N.A.) objective, or a Plan-Neofluar 40× objective (0.75 N.A.). Double-stained embryos (SYTOX orange and Phalloidin Alexa Fluor 488) were simultaneously excited at 488 nm and 543 nm and visualized on a FluoView FV1000 confocal microscope coupled to an up-right BX61WI Olympus microscope with a Plan FLN 10× (0.3 N.A.) objective or a Plan FLN 20× (0.75 N.A.) objective. The pinhole aperture was maintained at 105. Serial optical sections were obtained with a z-step of 8 µm. The images were processed with the public domain software ImageJ [26] the LSM Image Browser from Zeiss and Adobe Photoshop. To quantify the H3S10ph-positive nuclei in each embryo, focused images were made binary by thresholding to highlight in black the H3S10ph-positive nuclei and in white the background, and automatically quantified by the “analyze particles” command in ImageJ software. Embryo contour was delineated on each image, and the circularity was measured in Image J. The Student´s t-test was performed in Microsoft Excel for statistical analysis.

### 3.7. Immunofluorescence of α-Tubulin in PC3 Cells

PC3 cells were grown in RPMI medium supplemented with 10% FCS. In total, 3 × 10^4^ cells were cultured in 24-well culture plates containing slides and allowed to attach overnight at 37 °C in 5% CO_2_. The cells were then treated with compounds **1**–**7** at their IC_50_ values (0.95, 0.085, 1.0 × 10^−5^, 1.7 × 10^−3^, 2.0 × 10^−3^, 2.0 × 10^−3^, and 5.0 × 10^−3^ μg/mL, respectively) at 37 °C for 72 h. The cells were fixed with PFA (paraformaldehyde) 4% in PEM buffer (PIPES 100 mM pH 6.9, EGTA 5 mM, MgCl_2_ 2 mM). After 15 min, PFA/NaHCO_3_ was added, and the cells incubated for 45 min at room temperature. The slides were rinsed with PBS and treated with 0.1% Triton X-100 (Sigma Aldrich, Saint Louis, MO, USA), then incubated with a primary anti-α-tubulin antibody (1:300, Sigma Aldrich, Saint Louis, MO, USA) overnight at 4 °C. A secondary anti-mouse Alexa Fluor 647 antibody (1:1000, Molecular Probes, Eugene, OR, USA) was added, and the slides were incubated for 1 h at 37 °C. The cells were stained with 0.4 µg/mL of 4, 6-diamidino-2-phenylindole (DAPI, Molecular Probes, Eugene, OR, USA) in PBS for 10 min, mounted, and imaged by confocal microscopy as recently described [27].

## 4. Conclusions 

In this work, following a bioassay-guided fractionation by using the zebrafish model, we isolated seven antimitotic active lignans (**1**–**7**) from an antitumoral hydroalcoholic extract of *B. fagaroides.* We found by using immunoassays in zebrafish embryos that **1**, **2**, **4,** and **7** lignans induced mitotic arrest, delayed cell migration, and disrupted the microtubule cytoskeleton. These results enabled us to demonstrate that microtubule destabilization is the mechanism of action through which these compounds exert their cytotoxic activity.

Furthermore, microtubule array disruption was demonstrated also in PC3 cells. In general, equivalent antimitotic effects were observed in both in vivo and in vitro models.

Finally, we can conclude that the in vivo zebrafish model developed in this work is a suitable, faster, and cheap model to identify antimitotic natural products through bioassay-guided fractionation. 

## Supplementary Materials

Whole-mount immunolocalization of phospho-histone-H3 (H3S10ph) in zebrafish embryos. Table quantification of H3S10ph by the extract and fractions. ^1^H NMR table of pure compounds (**1**–**7**).
