# Supplementary material for: Lignans from Bursera fagaroides Affect In Vivo Cell Behavior by Disturbing the Tubulin Cytoskeleton in Zebrafish Embryos
Source: Molecules. 2018 Dec 20;24(1):8. doi: 10.3390/molecules24010008 (PMC6337621; doi:10.3390/molecules24010008)
Supplement: Supplementary file 1 [file molecules-24-00008-s001.pdf]

Supplementary Material

Lignans from *Bursera fagaroides* Affect the *In vivo* Cell Behavior by Disturbing the Tubulin Cytoskeleton in Zebrafish Embryos.

Mayra Antúnez-Mojica[1], Andrés M. Rojas-Sepúlveda[2], Mario A. Mendieta-Serrano[3], Leticia Gonzalez-Maya[4], Silvia Marquina[5], Enrique Salas-Vidal[3**] and Laura Alvarez[5,*]

[1]CONACYT-Centro de Investigaciones Químicas-IICBA, Universidad Autónoma del Estado de Morelos, Cuernavaca, Morelos, México.
[2] Facultad de Ciencias, Universidad Antonio Nariño, Armenia, Quindío, Colombia.
[3]Departamento de Genética del Desarrollo y Fisiología Molecular, Instituto de Biotecnología, Universidad Nacional Autónoma de México, Cuernavaca, Morelos, México.
[4]Facultad de Farmacia, Universidad Autónoma del Estado de Morelos, Cuernavaca, Morelos, México.
[5]Centro de Investigaciones Químicas-IICBA, Universidad Autónoma del Estado de Morelos, Cuernavaca, Morelos, México.

List of Supplementary material

Figure S1. Whole mount immunolocalization of phospho-histone-H3 (H3S10ph) in zebrafish embryos.
Figure S2. Fractionation tree diagram of fractions that were screened for their effect on the cell cycle and embryonic morphology in the zebrafish embryos by H3S10ph marker.
Table S1. Quantification of the effect of *B. fagaroides* HA extract, extract fractions an pure compounds on the cell cycle in zebrafish embryos.
Table S2. NMR ^{1}H of compounds **1-7**.

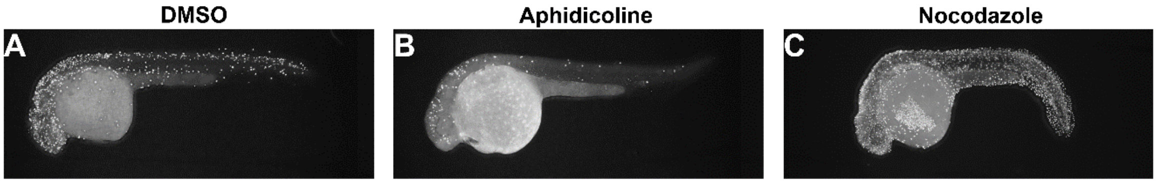

Figure S1. Whole mount immunolocalization of phospho-histone-H3 (H3S10ph) in zebrafish embryos. Wild-type 24-hour post-fertilization zebrafish embryos were immunostained after a 6-hour treatment with different compounds.

Table S1. Quantification of the effect of *B. fagaroides* HA extract, extract fractions and pure compounds on the cell cycle in zebrafish embryos

	ID	Cell cycle activity	H3S10ph fold change	*p* <0.001	n	N	t-student test
	DMSO		1.00 ± 0.32		23	3	
Drug	Aphidicoline	-	0.16 ± 0.09	*	27	3	1.54E-16
	Nocodazole	+	3.35 ± 1.18	*	30	3	1.73E-12
	Etoposide	=	0.59 ± 0.15		8	1	3.75E-02
Fraction	HA extract	=	1.10 ± 0.36		10	1	5.53E-01
	F-1	+	4.08 ± 0.97	*	9	1	2.40E-07
	F-2	+	4.48 ± 0.26	*	8	1	1.03E-14
	F-1-1	+	4.37 ± 0.72	*	9	1	1.38E-09
	F-1-2	+	4.28 ± 0.52	*	9	1	2.83E-11
	F-2-1	+	2.82 ± 0.99	*	10	1	1.03E-04
	F-2-2	+	4.21 ± 0.46	*	10	1	6.35E-11
	F-2-3	+	3.94 ± 0.74	*	9	1	1.07E-07
	1	+	2.95 ± 1.18	*	10	1	4.50E-04
	2	+	3.89 ± 0.97	*	9	1	2.00E-05
	3	=	0.79 ± 0.51		8	1	4.10E-01
	4	+	4.44 ± 0.99	*	8	1	5.43E-05
	5	=	0.76 ± 0.20		9	1	1.95E-01
	6	=	0.63 ± 0.29		10	1	5.85E-02
	7	+	2.88 ± 0.84	*	10	1	3.75E-05

ID, extract, fractions and compounds. H3S10ph, fold change compared with control DMSO-treated embryos. Cell cycle activity; -, denotes decrease, +, increase, =, without change. n, total number of embryos analyzed. N, number of experiments. Student's t-test, was used to determine the p-value.

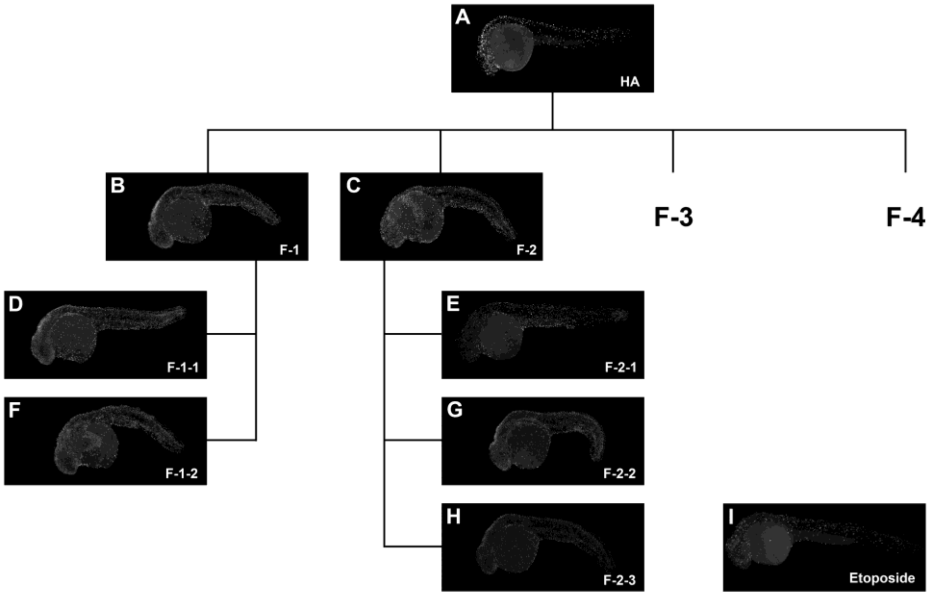

Figure S2. Whole mount immunolocalization of phospho-histone-H3 (H3S10ph) in zebrafish embryos. Fractionation tree diagram of fractions that were screened for their effect on the cell cycle and embryonic morphology in the zebrafish embryos. (A) Hydroalcoholic (HA) extract. (B) F-1. (C) F-2. (D) F-1-1. (E) F-2-1. (F) F-1-2. (G) F-2-2. (H) F-2-3. Etoposide. (I). Notice that in the present studies only fractions F-1, F-2 and their corresponding sub-fractions and pure compounds were included in the analysis. The other two fractions F-3 and F-4 are not included in the analysis of the present study.

Table S2. NMR ^{1}H of compounds **1-7**

	**1**	**2**	**3**	**4**	**5**	**6**	**7**
**Proton**	**δ 1H (ppm)**	**δ 1H (ppm)**	**δ 1H (ppm)**	**δ 1H (ppm)**	**δ 1H (ppm)**	**δ 1H (ppm)**	**δ 1H (ppm)**
**2**				6.44(d, 1H. *J*= 8)			
**3**	6.33(s, 1H)	6.20 (*s*, 1H)	6.255 (s, 1H)	6.45(d, 1H. *J*= 7.6)	6.50(s, 1H)	6.33(s, 1H)	6.48 (s, 1H)
**6**				6.43(s, 1H)	7.11(s, 1H)		6.72 (*s*, 1H)
**7β**	2.68 (*dd*, 1H)	3.11 (*dd*, 1H, *J*= 4.88)	2.36 (*dd*, 1H, *J*= 10.4, 16.8)	2.58 (*dd*, 1H)	2.86 (*m*, 1H)	2.68 (*dd*, 1H)	5.82 (d, 1H, *J* = 8.4)
**7α**		2.38 (*dd*, 1H, *J*= 10.8)	3.1 (*dd*, 1H, *J*= 4.8, 18)	2.50 (*dd*, 1H)	2.86 (*m*, 1H)		
**8**	2.96 (m, 2H, H-7, *J=3.2)	2.58 (m, 1H)	2.44 (m, 2H, H-7)	2.48 (m, 2H, H-7)	2.86 (*m*, 1H)	2.96 (m, 2H, H-7, J=3.2)	2.82(*m*, 1H)
**9α**	3.95 (*dd* 2H, *J*= 6.2)	3.86 (t, 2H, *J*= 10.4)	3.85 (*t*, 2H, *J*= 10)	4.12 (*dd* 2H, *J*= 7.2, 8.8)	4.076 (t 2H, *J*=10.8)	3.95 (*dd* 2H, *J*= 6.2)	4.16(*t*, 1H, *J*= 9.4)
**9β**	4.41 (*dd* 2H, *J*= 9.2)	4.40 (dd, 2H, *J*= 8.8)	4.37 (*t*, 2H, *J*= 6.8)	3.8 (m, 1H)	4.61(*dd*, 1H)	4.41 (*dd* 2H, *J*= 9.2)	4.34 (*dd*, 1H, *J*= 9.0, 6.2)
**2'**	6.59 (s, 1H)	6.80 (*s*, 1H)	6.96 (*d*, 1H, *J*=2.4)	6.66 (s, 1H)	6.37 (s, 1H)	6.59 (s, 1H)	6.33 (*s*, 2H)
**5'**	6.72 (d, 1H, *J*=8.4)		6.78 (*d*, 1H, *J*= 8.4)	6.67 (d, 1H, *J*=8)		6.72 (d, 1H, *J*=8.4)	
**6'**	6.60 (d, 1H, *J*=8)	6.80 (s, 1H)	6.36 (*dd*, 1H, *J*= 8)	6.68(d, 1H. *J*=7.6)	6.37 (s, 1H)	6.60 (d, 1H, *J*=8)	6.33 (*s*, 2H)
**7'**	4.33(d, 1H, J=2.8)	4.51 (*d*, 1H, *J*= 4.4)	4.51 (*d*, 1H, *J*= 4.4)	2.935(dd, 2H, 8´)	4.61(*dd*, 1H)	4.33(d, 1H, J=2.8)	4.54 (*d*, 1H, *J*= 4.0)
**8'**	3.25(dd, 2H, *J*=9.4)	2.618 (dd, 1H *J*= 13.6)	2.59 (m, 2H)	2.55(m, 3H, H-7´, H-8´)	2.86 (*m*, 1H)	3.25(dd, 2H, *J*=9.4)	2.82(*m*, 1H)
**O-CH$_2$-O (A)**	5.88(dd, 2H,)	5.84 (*s*, 2H)	5.89(*d*, 2H, *J*= 4.8)	5.93(d, 2H, *J*= 4)	5.97(d, 2H, *J*=1.2)	5.88(dd, 2H,)	5.93 (*d*, 2H, *J*= 1)
**O-CH$_2$-O (F)**	5.92(s, 2H,)					5.92(s, 2H)	
**<u>CH$_3$</u>CO**							2.13 (s, 3H)
**CH$_3$O-6**	3.92 (s, 1H)	3.99 (*s*, 3H)	3.99 (s, 1H)			3.92 (s, 1H)	
**CH$_3$O-3’**		3.68 (*s*,3H)	3.77 (*s*, 3H)	3.86 (*s*, 3H)	3.75 (*s*, 3H)		3.70 (*s*, 3H)

**CH_3O-5’**		3.68 (*s*,3H)			3.75 (*s*, 3H)		3.70 (*s*, 3H)
**CH_3O-4’**		3.73 (*s*, 3H)	3.74 (*s*, 3H)	3.84 (*s*, 3H)	3.80 (*s*, 3H)		3.75 (*s*, 3H)

**J = Hz*

**NMR spectra were acquired on a Varian Unity NMR spectrometer operating at 400 MHz. ^{1}H spectra were performed in $CDCl_3$ and referenced to Me_4Si (0 ppm)
